# Alternatives to the Use of Mechanical Restraints in the Management of Agitation or Aggressions of Psychiatric Patients: A Scoping Review

**DOI:** 10.3390/jcm9092791

**Published:** 2020-08-29

**Authors:** Damián Fernández-Costa, Juan Gómez-Salgado, Javier Fagundo-Rivera, Jorge Martín-Pereira, Blanca Prieto-Callejero, Juan Jesús García-Iglesias

**Affiliations:** 1 Faculty of Nursing, Department of Nursing, University of Huelva, 21071 Huelva, Spain; damianfernandezz98@gmail.com; 2 Faculty of Labour Sciences, Department of Sociology, Social Work and Public Health, University of Huelva, 21007 Huelva, Spain; juanjgarciaigl@gmail.com; 3 Safety and Health Postgraduate Programme, Universidad Espíritu Santo, Guayaquil 091650, Ecuador; 4 Andalusian Health Service, Primary Care Emergency Service, Health Sciences Doctorate School, University of Huelva, 21007 Huelva, Spain; javier.fagundo308@alu.uhu.es; 5 Hospital Transport Consortium, Isla Cristina Health Center, Isla Cristina, 21410 Huelva, Spain; jorgemppz@gmail.com; 6 Hospital Virgen de la Bella, Lepe, 21440 Huelva, Spain; blanca.prietoc@gmail.com

**Keywords:** agitation, aggressive behaviors, mental disorders, psychiatry, mechanical restraint

## Abstract

Coercive measures are a highly controversial issue in mental health. Although scientific evidence on their impact is limited, they are frequently used. Furthermore, they lead to a high number of ethical, legal, and clinical repercussions on both patients, and professionals and institutions. This review aims to assess the impact of the main alternative measures to prevent or limit the use of coercive measures with restraints in the management of agitated psychiatric patients. The research was conducted following the guidelines recommended by PRISMA (Preferred Reporting Items for Systematic Reviews and Meta-Analyses) in Medline, Cochrane Library, CINAHL, Web of Science, PsycInfo, LILACS, and Health Database of records between 2015 and 2020. After a critical reading, 21 valid articles were included. Both simple interventions and complex restraint programs were evaluated. Training in de-escalation techniques, risk assessment, and implementation of the “six core strategies” or “Safewards” program were the most assessed and effective interventions to reduce aggressive behaviors and the use of coercive measures. According to the revised literature, it is possible to reduce the use of restraints and coercive measures and not increase the number of incidents and violent behaviors among the patients through a non-invasive and non-pharmacological approach. However, further research and further randomized clinical trials are needed to compare the different alternatives and provide higher quality evidence.

## 1. Introduction

Psychomotor agitation can be defined as a feeling of agitation associated with an increase in motor activity caused by organic (stroke, seizures, sepsis, etc.) or psychiatric (derived from confusional states, psychotic or manic manifestations, effect or abstinence of substances, etc.) causes, which can quickly scale towards aggressive behaviors (ABs), considered to be any type of verbal aggression or threats, damage to property, to themselves, or to other patients or staff members [1]. Psychomotor agitation and aggressive behaviors are situations that can occur in any healthcare network service [2]. However, it is in mental health hospitalization units (MHHU) and psychiatric emergencies where psychomotor agitation or treating with aggressive or altered users is common [2,3].

Historically and generally in almost every developed country, the management of psychomotor agitation and AB is done through restrictive approaches, with the aim of controlling and reducing violent behaviors against the patient, others, or the environment [4,5]. This approach generally includes involuntary admittance and treatment, seclusion, and restraint [5]. Restraint explicitly refers to methods that limit freedom of movement, including physical restraints or mechanical restraint (MR), which is the method most widely used and, at the same time, the most controversial [4].

The term “mechanical restraint” refers to any method that includes application, control, and removal of mechanical fastening devices (belts, anklets, rails) used to directly limit physical mobility [5]. Although most experts agree on their indications of use, there are several ethical, legal, and clinical connotations to consider, and little data available to support the actual safety and efficiency of its use.

The use of restraints involves a practice of forced intervention that is at odds with the United Nations Convention on the Rights of Persons with Disabilities for limiting fundamental human rights such as freedom of choice or movement, autonomy, and physical integrity [6]. There is great variability regarding the clinical practice of these measures among countries, and even regions, subject to international, European, state, and local laws and regulations. This situation makes it difficult to develop guidelines for clinical practice, which would standardize, regulate, and control the use of measures and improve the transparency of recorded incidents and adverse effects in medical records and hospital documents. This situation causes arbitrariness and, above all, lack of control with regards to which type of technique to apply. In fact, statistics indicate that between 10% and 30% of patients admitted to MHHU in the United States and Europe are subjected to some form of coercive measures [2]. In 2003, the EUNOMIA project (European Evaluation of Coercion in Psychiatry and Harmonization of Best Clinical Practice), a multicentre study carried out in 12 European countries with the aim of developing a European Guideline containing relevant intervention, implementation, and control measures as a result of the non-existent regulation on coercive measures (CMs), showed large significant variations in the frequency of use among countries, being Poland, Greece, and Italy, the countries where these measures are more widely used [7].

Adverse effects of CMs, and especially MR, have repercussions for patients (physical and psychological injuries, decreased therapeutic partnership), health professionals (injuries, burnouts, diminished therapeutic alliance), and institutions (economic impact). Two recent studies have described a wide variety of physical and psychological effects for CMs [4,5]. Among all the existing ones, MR appeared to be the least tolerated as a variety of adverse effects (AE) (lacerations, rhabdomyolysis, bruises, etc.) have been reported for this sole practice, including death by strangulation or pulmonary embolism. Another inherent risk in this practice, even when a correct start-up and evaluation has been carried out according to the established protocols, is venous thromboembolism, with a prevalence of 1 in 10 patients, thus increasing the risk with the duration of the restraint [6]. Alternatively, seclusion and restraint (S/R) is associated with negative emotions, particularly with feelings of punishment and distress, highlighting the incidence of post-traumatic stress after S/R, which is between 25% and 47% of cases [5].

MR is considered to be a difficult and risky procedure, potentially dangerous for patients, but also to staff. Another recent study has suggested that MR involves unpleasant and stigmatizing practices for professionals, and does not exclude emotional discomfort and possible injuries during its application (kicks, strangulation, exposure to bodily fluids, etc.), which has caused many professionals to seek a justification for their use, not only for therapeutic purposes but also as punishment or to “educate patients” [8].

Since 1990, and especially in recent years, most guidelines and standards of regulatory agencies and accreditation bodies (The U.S. Centers for Medicaid & Medicare Services, The Joint Commission, The National Institute for Health and Care Excellence, etc.) recommend that MR should only be considered as a last resort, once all de-escalation techniques have failed and in the event of an emergency situation that compromises the life or physical integrity of the patient or third parties [2,3,4,5].

Given the high incidence of violent events among these types of patients and institutional efforts to reduce CMs, in 2012, a systematic review (SR) with meta-analysis offered by the National Institute for Health Research (NIHR) assessed a wide variety of pharmacological and psychosocial interventions with the aim of tackling this problem and identifying measures associated with a successful decrease in ABs. In fact, this review revealed significant new literature on the matter, and highlighted the scarcity of randomized clinical trials (RCTs) as compared with other fields of research, especially referring to non-pharmacological interventions and the need to improve the quality of the designs for future research [9].

On the one hand, two RCTs are highlighted for accumulating almost all the evidence regarding the successful decrease of ABs through individual alternative measures. Abderhalden et al., in 2008 [10], through a standardized evaluation of the risk of violence among hospitalized patients using the Broset Violence Checklisht (BVC), observed a decrease of 41% and of 27% for violent events and the use of CM, respectively, in intervention rooms. Van de Sande, in 2011 [11], compared a decrease in ABs and restraint rates through a daily risk of violence assessment by BVC and other predictive risk scales, and obtained a decrease in violent events in intervention rooms as compared with control rooms. In spite of the mainly positive results, in Abderhalden’s study, the severity of violent events did not decrease in a significant way by the end of the study. For Van de Sande’s RCT, after correcting for the number of days spent by the patients in the room, the decrease in the number of violent patients or the number of restraint measures used obtained significant statistics with respect to control rooms. In addition, the results could not be compared in the context of other evidences as these were the first obtained in those dates, nor could nursing staff be masked, who were in charge of assessing and predicting the risk of violence.

On the other hand, more recent SRs have suggested that many AB situations and the use of CM are preventable, through structured risk assessment, appropriate staff training in de-escalation techniques, and complex restraint programs that integrate various proactive non-invasive measures, based on scientific evidence, with fewer associated risks [2,4,5].

In this sense, it seems that scientific literature regarding RCTs on non-invasive and non-pharmacological interventions is still limited as compared with a great number of quasi-experimental studies that do not require randomization or informed consent, especially if the studies are focused on exclusive reduction of AB and RM [12,13].

On the basis of an exhaustive review of the literature, this study seems to be one of the first SR that aims to analyze the efficacy of alternative measures, with the purpose of preventing or reducing the use of RM separately on an agitated or violent psychiatric patient. At the same time, it aims at offering a more comprehensive view of the scope of these alternative measures. As a secondary objective, assessing the efficacy of these interventions was proposed to reduce the use of other coercive measures.

## 2. Experimental Section

A scoping review of the records of different databases was carried out, following an initially pre-established protocol that had been previously approved by the revision team in order to minimize the risk of publication and selection bias, thus ensuring optimal organization and content. This was done by applying the rules established in the PRISMA (Preferred Reporting Items for Systematic Reviews and Meta-Analyses) declaration [14]. Following the evidence-based clinical practice [15], and using the PICO format (see Table 1), a suitable question, which could be clinically answered, was prepared, and the different search strategies were derived from it, which are presented in Table 2.

The literature review was done between December 2019 and January 2020, in the following databases: Medline, Cochrane Library, CINAHL (Cumulative Index to Nursing and Allied Health Literature), Web of Science, PsycInfo, LILACS (Literatura Latinoamericana y del Caribe en Ciencias de la Salud), and Health Database, adapting the search terms to each meta-search engine. The studies search and selection were mainly carried out by the main researchers and scrutinized by a third researcher.

Although we originally intended to carry out a systematic review with a meta-analysis in order to assess the efficacy of alternatives to the use of MR, due to the lack of RCTs and given the great variability of designs in the field (see Section 4.3), this was not possible. Therefore, in order to meet the study objectives, other types of designs were included and a scoping review of the results found was eventually carried out.

Therefore, the following inclusion criteria were used: studies conducted between 2015 and 2020; scientific literature on clinical trials, analytical observational studies (cohort and control case studies), and quasi-experimental pre-post design studies to assess the efficacy of alternatives to coercive measures and systematic reviews to also gain a better understanding; written literature in English or Spanish; articles that discussed at least one of the following characteristics, i.e., simple interventions (Table 3) or complex S/R reduction programs, potentially useful in the reduction and prevention of MR use.

Opinion articles, case series, and records of low scientific evidence were excluded. Studies focusing on dementia patients were also excluded, as they often involve patients with different psychiatric and cognitive profiles for the interventions described here, which could deviate from the focus.

Once the studies were selected, a table was designed for the extraction of the following data: study type, country and date of publication, study design, sample size, evaluated measurements, efficacy, and methodological quality of the study. In order to better represent the references included, the table has been divided into primary studies and multiple-study reviews, stratified according to the level of methodological quality obtained (see Table 4) [16,17,18,19,20,21,22,23,24,25,26,27,28,29,30,31,32,33,34,35,36]. The impact of the analyzed interventions was determined by the results provided by the different studies in terms of a decrease in the number or mean time of use of mechanical restraints, or a decrease in the number of aggressive behaviors, duration, or severity (by validated aggression scales).

To evaluate the methodological quality of the study and ensure the selection of eligible articles, the web platform 3.0 for Critical Reading Sheets (FLC 3.0) was used [38]. This platform is a tool used for assessing the quality of a study and was developed by the Health Technology Assessment Service of the Department of Health of the Basque Government (Osteba) for systematic reviews, randomized clinical trials, and observational studies. It facilitates homogeneity among reviewers (Table 5), under the RedETS (Spanish Network of Health-Related Technology Assessment Agencies) [38]. The Effective Public Health Practice Project (EPHPP) “Quality Assessment Tool for Quantitative Studies” was used to assess the risk of bias of quasi-experimental studies [39] (Table 6). The EPHPP tool has been proven to be adequate for use in systematic reviews, it has a quite exact reliability for the reviewers, and an excellent degree of consensus for the final score. The EPHPP tool includes the following six categories (each containing 2 to 4 questions) to assess the study quality: (1) selection bias; (2) study design; (3) confounding factors, blinding; (4) data collection methods; (5) drop-outs or loses (6) [39].

Each category received a strong, moderate, or weak rating, which was the basis for the overall score of each study, i.e., 1, 2, 3, that correspond to a strong level of evidence (without any of the 6 weak categories), moderate (a weak category), and weak (two or more weak categories), respectively.

As a secondary strategy, a manual search was conducted on the relevant revision reference lists, on the essays included in those reviews, and their background articles to identify relevant references that our primary search might have be lacking. Both quality assessment and data extraction were carried out independently by two researchers, and a third researcher acted as assessor, comparing the data collected by both and concluding the contents of the final template. Any disagreement that arose among the researchers was resolved by discussion, and the third researcher’s opinion was sought when no consensus was reached.

## 3. Results

**Figure 1:**
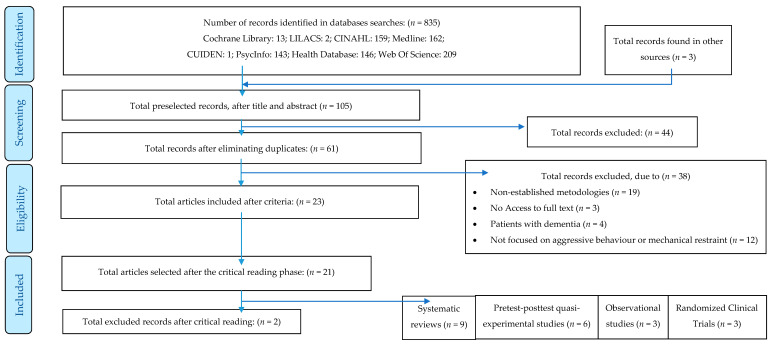
Preferred Reporting Items for Systematic Reviews and Meta-Analyses (PRISMA) flow chart for the process of selection of studies in systematic reviews (adapted from Moher et al. 2009 [14]).

A total of 105 studies was selected after a first screening (see Figure 1). After title and summary reading, studies that did not provide relevant data for this work were discarded. Among these studies, 61 studies prevailed after the removal of duplicates. Once assessed, those that met the pre-established inclusion criteria were selected, that is, a total of 23 records. One systematic review and a quasi-experimental study of pre-post design were excluded for showing low quality of evidence.

Following this process, and as happened in the last selection phase, 21 studies met the quality criteria established in the critical reading phase as follows: nine systematic reviews (three of which intended to conduct a clinical practice guideline based on their own literature reviews, and therefore are treated as such), three randomized clinical trials, three observational studies, and six quasi-experimental trials of interrupted time series.

Almost half of the studies included in this review were carried out in northern or central Europe (Norway, Denmark, Finland, Germany, etc.), although several articles were also developed in the United States and Canada. Most occurred in mental health hospitalization units for adults, in general or psychiatric hospitals, and some occurred in high-security or forensic psychiatry units. All studies were conducted on both men and women, except for the Long et al. [31] study on training in the “prevention and management of aggression and violence” (PMAV) program, which was carried out in an exclusively female service.

Since there were no previously agreed categories to stratify violence prevention and restrictive measures, all proposed alternatives to MR were divided into the following two broad blocks: clearly defined simple interventions (see Table 3) and complex S/R reduction programs, i.e., Omega Training Program, “Six Core Strategies” (6SC), “Safewards”, and PMAV.

In Table 4 [16,17,18,19,20,21,22,23,24,25,26,27,28,29,30,31,32,33,34,35,36], the main findings and aspects assessed from each of the included studies are shown, which included the following: author, date, type of study and country, proposed interventions, critical reading sheet used for the assessment and quality determination of the study, and main results of each of the included references.

According to data from this review, the events and duration of S/R (or coercive measures in those studies that assessed them separately from mechanical restraint) were the most common way to report on the decrease in the use of MR. When the impact of these measures was measured based on agitation or aggressive behaviors, it was done by the number of events, severity, and mean duration (measured by validated scales, usually OAS-M, BVC, and DASA).

## 4. Discussion

### 4.1. Simple Interventions

Training staff in the management of agitated patients is a priority, with a special preference for verbal de-escalation techniques. Five articles considered it to be the intervention of choice. According to a systematic review of 38 observational studies, a positive association was found between scale reduction and lower risk of violence or use of MR [32]. In the same line, another systematic review on alternative measures to S/R showed that nine out of the 13 assessed reviews recommend training staff, since a positive association between verbal de-escalation techniques and reducing coercive events in MR was found [27]. Moreover, an RCT on cluster sampling in the review by Vapikarta et al. [36] referenced that staff training was also related to a significant reduction in the average duration of use of CM (*p* < 0.05).

It should be noted that the three Clinical Practice Guidelines included in this review [17,21,32] with the aim of standardizing the management of acute psychiatric patients concluded the same thing, i.e., verbal de-escalation is the intervention of choice for the management of psychomotor agitation, due to its great ability to decrease the degree of anxiety and aggression. However, the evidence quality is low, and the results are inconclusive. In this sense, Patel et al. [32] and Baldacara et al. [17] attached a grade D recommendation based more on expert opinion and clinical experience than on high quality trials. Patel et al. [32] also included studying the evidence of each of the components that made up verbal de-escalation, although again most of them obtained only grade C or D recommendations, based on Evidence Level III: techniques of self-control, avoid provocation, respect patient space, empathy, and negotiation.

The scales are used to obtain an objective and standardized assessment of a patient’s escalation situation, and therefore the most appropriate measures for its management (2B) [17,21,32]. It appears that their use in combination with de-escalation techniques increases effectiveness in terms of reduction of MR (2C) [17].

The Brøset Violence Checklist (BVC) is a standardized tool for measuring the risk of aggressive behaviors and was effective in reducing the risk of violence and MR in a randomized clinical trial and the use of S/R in a systematic review, respectively. In the clinical trial by Hvidhjelm et al. [28] on the impact of BVC in 15 Mental Health Hospital Units in Denmark (2030 participants in total), BVC reduced the risk of violence by 45% as compared with the Clinical Guideline, and regarding the period of data collection and follow-up assessment (Phase 1–Phase 3). Despite this, the risk reduction was not statistically significant (OR-0.55, CI 0.21–1.43). Instead, it was in a randomized clinical trial of Hirsch and Steinert [27] systematic review where BCV significantly reduced the relative risk of restraints in the intervention group by 27%, while it increased by 10% in the control group (*p* < 0.001).

In this sense, another scale that measures the risk of violence and seems to be successful when combined with de-escalating interventions is the DASA. Kaunomaki et al. [29], in their prospective longitudinal study, noted that of the DASA >4 screenings (indicates a high risk of violence), 91.2% followed treatment, mainly PRN (“pro re nata” medication, i.e., on-demand medication, as needed) but only with “other interventions” such as verbal application of limits or change of medication was there a significant association with a lower total DASA score the next day (*p* < 0.001).

In addition, the record of the number of adverse events (AE) related to MR or any other restraint method and the analysis made by the team members (debriefing) offered a broader knowledge of these events and helped reduce them in the future [37]. Several scales that assess the risk of AB among patients also seem useful in recording clinical trials, for instance, BVC and DASA [28,29], as well as the OAS scale in the RCT by Nurenberg et al. [30] on animal-assisted therapies (AAT), or the PCC scale [19] in the RCT on the “Safewards” program by Bowers et al., which recorded 3403 conflict events between patient and staff member during the study phase.

Two articles [30,33] showed that the inclusion of hospitalized patients with acute metal disorders in psychosocial or behavioral therapies are well tolerated and related to an improvement in their behaviors in the short and medium term, as well as fewer violent incidents. In this sense, the randomized clinical trial on 90 participants by Nurenberg et al. [30], which evaluated different modalities of animal-assisted therapy (“equine-assisted therapy” (EAT) and “can-assisted therapy” (CAT)) and a “skills group psychotherapy” (SSP) in a U.S. psychiatric hospital, showed that the three interventions showed significant differences regarding the control group (*p* = 0.035), and that aggression against objects (*p* = 0.005) and people (*p* = 0.0053) decreased. However, reports of violent incidents within the different intervention groups suggested a decrease only in EAT patients, which were reduced as compared with increased or unchanged levels for other groups (*p* = 0.029 versus CAT for objects, *p* = 0.074 versus SSP for people). This article was rated, along with 22 other articles, in the systematic review by Rampling et al. [33], which studied a wide range of psychological and psychosocial therapies to reduce violence (physical, verbal, or violent attitude) in patients with acute metal disorder, including even some specific programs for psychosis, i.e., the Cognitive Behavioral Therapy for psychosis in male patients with a history of psychotic violence, that showed a significant reduction of aggressions, and therefore less chance of having to resort to MR. However, the RCT by Nurenberg et al. [30] on AAT was the most efficient psychotherapeutic program for aggressive behavior reduction.

Engaging patients in their own care (medication, preferences, how to address it in the event of a crisis, and taking into account their preferences) has been linked to a better therapeutic relationship and lower rates of violent incidents and use of MR. In the study by Kaunomaki et al. [29] on the effectiveness of DASA in measuring the risk of violence, “discussion with a nurse” (empathic listening, active communication, conscious presence, touch, etc.) helped focus on the reasons for aggressive behavior and facilitate better ways to treat these problems in the room. However, its use was not reflected as statistically significant differences in the DASA score the next day (*p* = 0.34). In the SR on effective measures to reduce the use of MR by Vapikarta et al. [36], some authors found an association between “patient involvement” and a reduction in MR, although these results contradicted other articles from the same review which found no differences.

Despite all these measures, the management of psychiatric patients is often still a difficult task, especially if excitation factors are beyond the control of professionals. In fact, the literature suggested a positive association between excessive sensory stimuli (intrinsic or extrinsic) and feelings of insecurity and loss of control by patients with MD, closely related to schizophrenia and bipolar disorder, which often led to the use of MR [16,17]. Four studies identified different sensory modulation (SM) programs as an effective therapeutic approach for distressed, agitated, and potentially violent patients [16,25,27,36]. The case-control study by Andersen et al. [16] on two units of a Danish psychiatric hospital assessed the relationship between SM and a decrease in S/R and MR alone. In the case room, a risk assessment was carried out using BVC and ASP (“Adult Assessment Profile”) for sensory processes. Then, depending on the ASP, occupational therapists, in collaboration with nurses (after training the staff in SM activities), created an individual or group plan consisting of SM activities (recreational activities, learning to self-regulate, relaxing music, skills to voluntarily steer away from stress or stimuli overload, and sensory room for those most at risk of excitement). Through the use of SM, S/R was significantly reduced by 42% in the case room with respect to the control room, OR = 0.58 (0.38–0.90). Although when MR was measured alone, it was reduced by 38%, but without finding statistical significance, OR 0.62 (0.34–1.13). Four controlled clinical trials included in the systematic review by Hirsch and Steinert [27] also showed a reduction in S/R, provided that continuous nursing care and therapeutic instruction were offered. However, in the systematic review by Vapikarta et al. [36], the results of studies between “patient involvement and SM” and MR reduction were contradictory.

SM and a better therapeutic environment are highly related. It is recommended that the rooms have adequate climate control and minimum external stimuli such as temperature, light, noise, and ventilation, although again these recommendations are mostly based on clinical experience due to the lack of empirical evidence (D) [17,27].

Finally, the organizational impact of the hospital door policy, the type of room, and the number of rooms and patients per floor influences the therapeutic environment and the use of S/R. Schneeberger et al. [34] conducted a retrospective 15-year follow-up cohort study (1998–2012) on 21 German psychiatric hospitals (16 closed, four open, and one open until 2010), to assess the impact of open door policy and open room policy regarding aggressive behaviors and the use of S/R. In total, during the study period, data were collected from 314,330 patients (246,195 for the closed door policy, 68,135 for the open door policy, and 63,114 patients were included per group for the matching data analysis). The open door policy showed a statistically significant impact on the reduction of MR on the matching dataset, OR 0.36 (0.19–0.69), ratified in the unmatched analysis, OR-0.48 (0.25–0.91); but without significant differences for aggressive behavior. For its part, the open room policy did show a significant impact on the reduction of aggressive events, both in the paired design and in the unmatched analysis, OR 0.10 (0.08–0.14) and OR-0.13 (0.10–0.16), respectively; also, for reducing the use of restrictions. OR 0.27 (0.17–0.41) and OR 0.24 (0.16–0.36), respectively.

### 4.2. Complex Programs

In this review, greater empirical evidence and the impact of complex programs were found with respect to simple interventions in terms of S/R and aggressive behaviors reduction; several articles highlighted their greater evidence. For example, in the systematic review by Hirsch and Steinert [27], 37 of 38 articles that assessed complex programs reported a reduction in MR rates and their impact according to randomized clinical trials that assessed them (Safewards, 6CS, and “engagement model”, the latter not being analyzed by other studies included in this review). Moreover, a systematic review of 28 articles and another of 21 articles [22,36] found that only the implementation of a reduction program gave mainly positive results in several studies, or that only multimodal and “risk assessment” programs were associated with fewer violent episodes and MR, respectively. These results are in line with what is found in the scientific literature [24].

The Omega Training program emerged in Canada, in 1999, given the need for greater staff training to prevent violence in the workplace [2,23,25]. One of the studies on which its impact was based was carried out at Institut Universitaire en Santé Mentale de Montréal (IUSMM), one of the two main institutes dedicated to mental health in the city of Montreal, Canada, given the high number of aggressions towards professionals in this field. Guay et al. [25], through their quasi-experimental study, showed that there were statistically significant differences regarding before and after Omega Training in terms of major and minor violence reductions and greater confidence in dealing with aggression (*p* < 0.05), except for the “pressure” variable, which suffered no changes (*p* = 0.20). Geoffrion et al. [23], through an interrupted time series analysis, decided to check whether the Omega Training program had an impact on reducing aggressive behaviors and S/R in emergencies (880 S/R events throughout the study) and psychiatric intensive care units (6993 S/R events). The resulting observations were a significant reduction in the use and duration of MR in intensive care units during and after training (*p* = 0.0157, *p* = 0.035, for the number of MRs, respectively), although without finding differences for the emergency room (*p* = 0.19, *p* = 0.20, for the number of MRs, respectively), which they attributed to the constant volume of admissions and discharges, and to the intense activity in this plant that makes nurses less likely to forge a therapeutic relationship.

For the PMAV multimodal program, only one pretest-posttest trial was found, and therefore the evidence extracted in this review is limited. Long et al. [31], by training nurses in PMAV based on the use of verbal de-escalation and prevention and involvement of patients in the management of risky behaviors, found significant differences only in decreased isolation between the two study periods (*p* < 0.01). Although there were no significant differences in the number of MRs, there were regarding the duration of MR (27 min to 7 in period 2, *p* < 0.01) and in the aggressive behavior rate towards objects or others, using the OAS (*p* = 0.034 and 0.043, respectively), which indirectly affected the frequency of MR use.

The 6CS reduction program and its adaptations is the most widely used and covers the greatest amount of evidence available. Originally from the USA, it has been adopted and studied by different hospitals and rooms around the world (Finland, Australia, UK, and Spain) [24]. This review included four studies individually evaluating the “Six Core Strategy”. Seven studies in the review by Hirsch and Steinert [27] showed a positive association between the implementation of 6CS and a reduction of S/R and aggressive behavior. Framed within this systematic review and also included in the Clinical Practice Guidelines by Patel et al. [32], the “cluster-randomized controlled trial of reducing seclusion and restraint in secured care of men with schizophrenia” was the first RCT to find a 30% to 15% reduction in S/R in the intervention rooms as compared with the control rooms that did not implement the 6CS (25–19%) (*p* < 0.001). Thus, this clinical practice guide suggests its implementation. For his part, Guzman-Parra et al. [26], in their study on the impact of an adaptation of the 6CS to the Spanish health environment, found that the likelihood of using mechanical restraints in a hospital admission significantly decreased, OR = 0.58 (0.41–0.83). A reduction in the number of total MR hours was also found given the lower number of MRs used. Thus, the authors recommended the use of this scale in the context of Spanish psychiatry, although these results should be taken with caution, as no further studies to date were found that can contrast this information.

Of all the complex programs evaluated in this systematic review, “Safewards” is the most recent, but also the most promising given its strong theoretical evidence after a review of more than 1000 external studies [40]. It has also obtained a good assessment in this systematic review. “The Safewards cluster randomized controlled trial” by Bowers et al. [19] was a RCT which analyzed the effectiveness of the package of 10 interventions proposed by Safewards to reduce conflict rates (according to the authors: verbal aggression, suicide attempts, alcohol use, escape attempts, etc.) and containment measures (forced medication, isolation, and physical restraint) in 31 psychiatric wards of 15 London hospitals. The study showed the following very promising and generalized results: In the 16 intervention rooms, the measurement achieved a significant 15% reduction in conflict rates (CI 95% 5.6–23.7%) and 24.6% in restraint rates (CI 95% = 9.9–34.3%) as compared with control rooms. Safewards was also very effective for reducing isolation in a controlled clinical trial of 44 MHHUs in Australia, as assessed by Hirsch and Steinert [27].

As the use of MR is not contemplated in English psychiatry, this review includes two pretest-posttest studies to analyze its effectiveness with respect to MR and other environments [28,35]. Stensgaard et al. [35], found that a significant increase in the rate of CM use decreased (these were already decreasing before the implementation thanks to institutional efforts) from 1% to 3% following the implementation of “Safewards” (*p* < 0.001, CI 95% 2–5%), a decrease of 4% in the use of MR per trimester (*p* < 0.001, CI 95%: 3–5%) remained without differences after the program implementation (*p* = 0.4). Moreover, the study by Baumgardt et al. [18] (Germany) did not find significant differences in the use of MR either, in any of the study’s two MHHUs (A and B), but only a significant reduction in the event room B and in the overall duration of CM (*p* = 0.03 and *p* = 0.032, respectively).

In general, most of the analyzed interventions have shown favourable results, as long as their objectives have been the reduction of aggressive behavior and MR in general. Unfortunately, reducing restraints was not always possible, since most studies included them in all the CMs used, or those in which these were analyzed separately did not obtain statistical significance or the results between studies were not homogeneous. For example, in the case-control by Andersen et al. [16] on SM, the intervention decreased overall S/R by 42%, and MR by 38%, although without finding significant differences for the latter, OR 0.62 (0.34–1.13). In the same line, in the study by Hvidhjelm et al. [28], the use of BVC (as a predictor for patients at risk of violence) in combination with de-escalation techniques showed a 45% reduction in violence, and MR was only used in 2.4% of all interventions. This study did not find significant differences regarding the control group either. It was also in contradiction with other studies framed in this review [36].

In this review, among all the individual interventions studied, verbal de-escalation techniques and risk assessment for the prediction of patients at risk of violence and reduction of MR have been highlighted, recommending their teaching and training to health professionals, and especially to MH nurses.

The combination of different measurements optimizes the efficiency that these would obtain separately, for example, using the BVC or DASA scale together with de-escalation techniques or “discussion with a nurse” [28,29], OAS-M and animal-assisted therapy [30], BVC together with ASP in combination with sensory modulation [16], or open door and open room policies along with decreased environmental stimuli [17,27,34]. It would also be interesting to inform patients about these therapies, as well as to involve them and their families in their care and responsibilities.

Therefore, it is believed that the use of complex S/R reduction programs that integrate several partial measures at the same time are those that best meet the study objectives.

The results of this study add to the “Safewards” model evidence base. This review has shown, on several occasions, its great ability to decrease aggressive behaviors and the use of S/R [19], and even to reduce isolation [27]. However, since MR is not used in English psychiatry, it is again not possible to separately assess a potential reduction in MR, and when trying to implement this in other countries where its use is contemplated, unfortunately, “Safewards” shows no effectiveness for its reduction [28,35].

### 4.3. Limitations

One possible study limitation was to ignore written literature in languages other than those set out in the inclusion criteria. Another possible limitation is the criteria of 5 years back into the past, that is generally more extensive for this type of review. However, the review team considered it valid under consensus, because, before publication in 2015 of “The Safewards cluster randomized controlled trial” [19], there were no previous RCTs on interventions to reduce AB or the use of coercive measures as a whole. Even regarding individual AB or restraint events (different CM separately), few RCTs have been developed. However, RCTs by Abderhalden [10] and Van de Sande [11] are still today of great impact on the dissemination of risk of violence structured assessment in the short term, not only to predict but also to be used as effective measures as regards CM. Finally, their results suggest that risk of violence assessment cannot imply, by themselves, an improvement in the clinical practice and, unfortunately, they do not either assess reducing MR exclusively, but they do regarding AB.

Despite the efforts made to obtain high-quality methodological trials, only five studies with control groups were included because, unlike studies with pharmacological interventions, blinding of staff is not possible, nor it is their formal randomization in clinical services environments that imply individual contact, as is the case of psychological and psychosocial therapeutic interventions. It is not possible either, many times, due to ethical, legal, and practical reasons, to obtain the informed consent of patients who are temporarily or indefinitely unable to decide whether or not to participate in a clinical trial [9]. Thus, the need for studies with these characteristics is stated by most authors [24,27].

Thus, a large part of the studies included were quasi-experimental pretest-posttest studies based on observational studies and often retrospective designs without the need for consent from participants, and systematic reviews that highlighted the same limitation [24,27,33]. Due to the lack of control groups in these types of studies, several denoted some uncertainty regarding the actual cause of MR or aggressive behaviors reduction beyond the suggested intervention, which may possibly affect the generalisation of this study results.

In order not to draw biased conclusions, the following can be highlighted: being aware of the risk of bias in pretest-posttest design studies, the results were analyzed with great caution and as long as it was possible to contrast the information with other higher quality studies. Similarly, in order to avoid an overestimation of the effects of the interventions, both the results that found a decrease in the use of MR or de-escalation of aggressive behaviors and those that did not achieve significant differences were considered.

### 4.4. Relevance for Clinical Practice

On the basis of the findings of this study, we recommend adopting organizational strategies that promote a less restrictive and safer care policy and care culture, that reward teamwork, and that strengthen the patient-professional therapeutic alliance. In addition, health professionals should be aware and trained, especially nurses due to their closest contact with patients, and therefore being the most likely to benefit from these improvements.

## 5. Conclusions

On the basis of the revised literature for the reduction of violent incidents and the use of MR in different mental health areas, the following conclusions are drawn: The approach towards an agitated or violent patient should begin with less coercive measures. Early detection through validated scales in order to conduct an objective risk assessment is essential in this regard in order to enhance less invasive measures. Staff training in verbal and non-verbal de-escalation techniques is recommended as a treatment of choice over MR. As preventive measures, the use of psychotherapies (AAT, CBT), the modulation of sensory stimuli, and the active participation of the patient in their own care, together with an analysis and compilation of adverse events, shows hopeful results regarding an overall reduction of coercive measures and better knowledge and awareness about their use. Since many of these measures are often part of more complex reduction programs, it is recommended that institutions develop and put them in place for their implementation.

The findings of this study broadly agree with the scientific literature, which emphasize that a combination of interventions to minimize the use of CM is necessary both individually and in terms of the involved organization, as well as the adoption of complex highly evidenced models [17,21,22,24,27,32,36,41].

In addition, the results of this study are in line with most experts on the matter who highlight the dramatic lack of RCTs that determine the actual efficacy of alternative measures to the use of MR and, similar to this study, encourage the urgent need for further research with greater methodological rigour [9,10,11,12,13,19,20,22,24,25,33,42].

The goal of “reducing force in mental health care with the help of guidelines” is the objective of a large-scale multicentre group RCT currently in process, which, since 2019, is being held at 52 German MHHUs is precisely the one stated above [42]. From a list of 12 interventions, of which the vast majority have previously been evaluated by the literature and in this review (risk assessment via BVC, standardized registration of adverse events on CM, training in de-escalation techniques, patient participation, implementation in the 6CS room, Safewards, etc.), intervention rooms should choose two or three of these with the aim of successfully reducing the use of CM (MR, isolation, physical restriction, and forced medication), without an increase in the number of violent incidents due to the reduction in the use of these measures. Many hopes have been placed on this study so that it can finally provide better technical scientific standards and the correct use of alternative coercive measures for the management of agitated and violent psychiatric patients in the very near future.

## Figures and Tables

**Table 1:** Structure of the research question through the population, intervention, comparison, and outcomes (PICO) format.

Population	Agitated psychiatric patients
Intervention	Strategies to prevent agitation, aggressive behaviour, and/or use of MR
Comparison	Usual treatment and/or coercive measures
Outcomes	Prevent or reduce escalation towards aggressive behaviours or the use of CM (specially MR) in mental health.

CM, coercive measures, MR, mechanical restraints.

**Table 2:** Search strategy in databases.

Database	Date	Search Strategy	TA	PA
LILACS	20 June 2020 18:00	#1(Mental disorders [MH] OR psychiatry OR psychiatric) #2 (coercion OR restraint OR mechanical restraint) #3 (psychomotor agitation [MH] OR aggressive behavior OR aggression [MH]) #4 #1 AND #2 AND #3 AND la:(“en”)) AND (year_cluster:[2015 TO 2020])	2	1
	20 June 2020 10:17	#1 (Trastorno mental [DeCS] OR psiquiatría OR psiquiátrico) #2 (coerción OR restricción OR contención mecáncia) #3 (agitación psicomotriz [DeCS] OR comportamiento agresivo OR agresión [DeCS]) #4 #1 AND #2 AND #3 AND la:(“en”)) AND (year_cluster:[2015 TO 2020])	0	0
CUIDEN	20 June 2020 12:33	#1(Mental disorders [MH] OR psychiatry OR psychiatric) #2 (coercion OR restraint OR mechanical restraint) #3 (psychomotor agitation [MH] OR aggressive behavior OR aggression [MH]) #4 #1 AND #2 AND #3	1	0
	20 June 2020 17:40	((“Trastorno”) AND ((“mental”) AND ((““) OR ((“psiquiatría”) OR (“psiquiátrico”))))) AND (((“coerción”) OR ((“restricción”) OR ((“contención”) AND (“mecáncia”)))) AND ((“agitación”) AND ((“psicomotriz [DeCS]”) OR ((“comportamiento”) AND ((“agresivo”) OR (“agresión [DeCS]”))))))	0	0
Cochrane Library	20 June 2020 11:44	#1 MeSH terms: [Mental Disorder] explode all trees #2 MeSH terms: [Mental Health Services] explode all trees #3(severe mental disorder* OR severe mental illness* OR psychiatry* OR psychiatric*) (search word variations) #4 #1 AND #2 AND #3 #5 (non-pharmacological intervention OR reduc* OR prevent*) (search word variations) #6MeSH terms: [Restraint, Physical] explode all trees #7(coercion OR restraint OR coerciv* OR mechanical restraint) (search word variations) #8 #4 AND #5 AND #6 AND #7 #9 MeSH terms: [Psychomotor Agitation] explode all trees #10 MeSH terms: [Aggression] explode all trees #11 #8 AND #9 AND #10 #12 (aggress* OR aggressive behavior OR violent) (search word variations) #13 #4 AND #11 AND #12 Publication year between 2015 and 2020	13	6
CINALH	20 June 2020 11:35	#1 (mental disorder OR severe mental disorder* OR severe mental illness* psychiatry* OR psychiatric* OR “Mental health hervices”) #2 (non-pharmacological intervention OR reduc* OR prevent*) #3(coercion OR restraint OR coerciv* OR mechanical restraint) #4(psychomotor agitation OR aggressive behavior OR aggression OR violent* OR “violence”) #5 #1 AND #2 AND #3 AND #4 Limits—Publication date: 20150101-20201231; Expanders—Apply related words; Apply equivalent subjects; Specify by Language: English; Spanish; Search modes—Boolean/Phrase	104	17
	2 January 2020 09:53	#1 (mental disorder [MH] OR severe mental disorder* OR severe mental illness* psychiatry* OR psychiatric* OR “Mental health hervices”[MH]) #2 (non-pharmacological intervention OR reduc* OR prevent*) #3(coercion OR restraint OR coerciv* OR mechanical restraint) #4(psychomotor agitation [MH] OR aggressive behavior OR aggression [MH] OR violent* OR “violence” [MH]) #5 #1 AND #2 AND #3 AND #4 Limits—Publication date: 20150101-20201231; Expanders—Apply related words; Apply equivalent subjects; Specify by Language: English; Spanish; Search modes—Boolean/Phrase	55	0
MEDLINE [via Pubmed]	4 January 2020 16:44	(mental disorders OR severe mental disorder* OR severe mental illness* psychiatry* OR psychiatric* OR “Mental Health Services”) AND (non-pharmacological intervention OR reduc* OR prevent*) AND (coercion OR restraint OR coerciv* OR mechanical restraint) AND (Psychomotor agitation OR aggressive behavior OR aggression* OR violent OR violence) Filters applied: complete text, in the last 5 years, English, Spanish	162	27
Web of Science	8 January 2020 17:22	(mental disorders OR severe mental disorder* OR severe mental illness* psychiatry* OR psychiatric* OR “Mental Health Services”) AND (non-pharmacological intervention OR reduc* OR prevent*) AND (coercion OR restraint OR coerciv* OR mechanical restraint) AND (Psychomotor agitation OR aggressive behavior OR aggression* OR violent OR violence) Refined by: AND LANGUAGES: (ENGLISH OR SPANISH) Time lapse: Last 5 years	160	23
	12 January 2020 11:39	(((mental disorder[MH] OR severe mental disorder* OR severe mental illness* psychiatry* OR psychiatric* OR “Mental health hervices”[MH])) AND ((non-pharmacological intervention OR reduc* OR prevent*)) AND ((coercion OR restraint OR coerciv* OR mechanical restraint)) AND ((psychomotor agitation[MH] OR aggressive behavior OR aggression [MH] OR violent* OR “violence”[MH]))) Refined by: LANGUAGES: (ENGLISH) Time lapse: Last 5 years	49	0
Health Database	13 January 2020 17:06	(mental disorders OR severe mental disorder* OR severe mental illness* psychiatry* OR psychiatric* OR “Mental Health Services”) AND (non-pharmacological intervention OR reduc* OR prevent*) AND (coercion OR restraint OR coerciv* OR mechanical restraint) AND (Psychomotor agitation OR aggressive behavior OR aggression* OR violent OR violence) Filters applied: complete text, in the last 5 years, English, Spanish	146	7
PsycINFO [via ProQuest]	19 January 2020 20:53	(mental disorders OR severe mental disorder* OR severe mental illness* psychiatry* OR psychiatric* OR “Mental Health Services”) AND (non-pharmacological intervention OR reduc* OR prevent*) AND (coercion OR restraint OR coerciv* OR mechanical restraint) AND (Psychomotor agitation OR aggressive behavior OR aggression* OR violent OR violence) Additional limits—Date: From 1 January 2015 to 1 January 2020; Language: Spanish, English	143	24
Total articles found, 835				
Total preselected articles, 105				
Total articles after duplicates removal, 61				

TA, total articles; PA, preselected articles.

**Table 3:** Identified simple interventions and their correspondence with 6CS (*Six Core Strategies* to reduce seclusion and restraint use) [37].

Category	6CS Category	Definition	Adopted Measures
Staff training	Workforce development	Promoting training and improvement in knowledge and non-invasive skills to the professionals involved in the management of agitated or aggressive consumers.	Verbal de-escalation techniques
Risk assessment	Use of S/R prevention tools	Using assessment tools to identify risk of violence and persons at risk of escalating to aggressive behaviors or trauma; to guide in reducing escalation; to use a universal trauma assessment; etc.	Risk of agitation: OAS, OASS, ASP Risk of violence: BVC, DASA
Use of data shared by post-S/R	“Use of data to inform practice” and “questioning strategies”	Monitoring, collection, and use of data to identify the facility/units’ S/R use baseline in adverse events, the number of involuntary medications, and the consumer and staff demographic characteristics. It includes the tracking of injuries related to S/R events to obtain further knowledge and try to reduce them.	“Debriefing”, “associated risks revision”, PCC, AOS
Patient involvement	Consumer roles in in-patient settings	Full and formal inclusion of consumers and relatives in their care, and participation in organisational decision-making (in their psychiatric hospital unit).	“Discussion with a nurse”, “establishing limits”, “discussing measures with the consumer”, psychotherapies
Therapeutic environment	Leadership towards organizational change	Promoting a safe environment with the sufficient stimuli for consumer self-management, promoting ability to participate without being overly stressed, coerced, or overwhelmed, and reducing anxiety, agitation, or insomnia.	SM, reducing environmental stimuli
Organizational changes	Leadership towards organizational change	Defining a philosophy of care and assuring for the development of a S/R reduction plan through the guidance, direction, participation, and ongoing review by executive leadership.	ODP, ORP, allowing permits to patients

6CS, Six Core Strategies; AOS, Aggression Observation Short Form; ASP, Adult Assessment Profile; BVC, Broset Violence Checklist; DASA, Dynamic Appraisal of Situational Aggression; OAS, Overt Aggression Scale; OASS, Overt Agitation Severity Scale; SM, Sensory Modulation; ODP, open doors policy; ORP, open room policy; PCC, patient-staff conflict checklist; S/R, seclusion and restraint.

**Table 4:** Characteristics of the included studies.

Author	Country and Date	Study Design	Participants	Evaluated Measures	Efficacy	Quality/Grade of Recommendation
**Primary studies**						
Andersen C. [16]	Denmark 2017	Case-Control	218 subjects	Sensory modulation (SM)	Sensory modulation (SM) was useful in reducing the use of Mechanical Restraints (MR) (OR = 0.58 (0.38–0.90), while not specifically for Coercive Measures (CM) (OR = 0.62 (0.34–1.13)	High
Baumgardt J. [18]	Germany 2019	Quasi-experimental study	103 subjects	“Safewards”	“Safewards” was effective in reducing S/R in general (*p* < 0.003), but there were no differences in terms of CM (*p* > 0.05)	Medium *
Bowers L. [19]	United Kingdom 2015	RCT	31 MMHU: 16 from IG and 15 from CG.	“Safewards”	“Safewards” reduced conflict events in 15% (CI 95%: 5.6–23.7%) and S/R events in 26.4% (CI 95%: 9.9–34.3%)	High
Geoffrion S. [23]	Canada 2018	Quasi experimental study	6993 events of S/R in ICU, and 880 in Emergencies	“Omega”	“*Omega*” was not related to a decrease in S/R during the three periods of measurement (*p* = 0.19)	Medium *
Guay S. [25]	Canada 2016	Quasi experimental study	105 subjects	“Omega”	“*Omega*” has a significant impact on psychological distress, safety perception, and level of exposure to aggressive behaviors for health staff, so they can more adequately respond to violent patients (*p* < 0.05)	Medium *
Guzman-Parra J. [26]	Spain 2015	Quasi experimental study	1575 subjects (735 pre-intervention and 840 during intervention)	“Six core strategies”	“Six core strategies” was effective in significantly reducing the probability of using MR during hospital admittance [OR = 0.58 (0.41–0.83)]	Medium *
Hvidhjelm J. [28]	Denmark 2016	RCT	2030 subjects	Risk scales (BVC), verbal de-escalation	Risks assessment reduced the risk of violence in the intervention group in 45% for scores >1 and was efficient in reducing the use of MR (2.4%). However, it did not obtain statistical significance (OR = 0.55, CI 0.21–1.43).	High
Kaunomaki J. [29]	Finland 2017	Observational prospective study	300 subjects	Risk scale (DASA), “Discussion with a nurse” (empathic listening and active communication), verbal establishment of limits	After assessing patients’ risk of violence, only non-coercive measures obtained a lower total DASA score the following day (*p* < 0.001)	Medium
Nurenberg J.R. [30]	USA 2015	RCT	90 subjects	Psychosocial therapies (AAT: EAP, CAP, SSP)	In the intervention group, AAT, and specifically, EAP, showed decreased violence as compared to others (*p* < 0.053), and against objects (*p* < 0.05). However, no differences were found in reducing CM	High
Long, C. [31]	United Kingdom 2016	Quasi experimental study	124 subjects	PMAV	PMAV did not show statistically significant differences in reducing MR, though it did regarding their mean duration between t0 and t1 (*p* < 0.01). PMAV reduced aggressive behavior against others and objects (*p* = 0.034 and 0.043, respectively). No differences were found regarding verbal aggression and self-injury.	Medium *
Schneeberger, A.R. [34]	Germany 2017	Cohort study	314,330 subjects	Organizational changes (Open Door Policy, Open Room Policy)	Both Open Door Policy (OR = 0.36 (0.19–0.69) and Open Room Policy (OR = 0.27 (0.17–0.41) are efficient in reducing MR. Open Room Policy is also efficient in reducing aggressive behavior, OR = 0.10 (0.08–0.14).	Medium
Stensgaard L. [35]	Denmark 2018	Quasi experimental study	12,600 events S/R; 2948 mechanical restraints	“Safewards”	“*Safewards*” did not show significant changes (*p* = 0.4) in decreasing rates of MR usage, which were already decreasing thanks to institutional efforts. However, this was effective in reducing S/R events.	
**Multiple-study reviews**						
Baldacara, L. [17]	Brazil 2019	Clinical Practice Guidelines	104 subjects	Therapeutic environment of the team, risk scales, verbal de-escalation, RT.	The use of scales allows an objective and standardized assessment of the escalation situation of the patient and the appropriate measures for its management (2B). The chosen intervention to manage acute agitation so as to calm the patient includes verbal de-escalation techniques (D)	High
Dahm, K.T. [20]	Norway 2017	Systematic review	21 studies	ACT, risk assessment, staff training, and advanced instructions at discharge	According to a randomized clinical trial (RCT), the joint use of risk scales and team training techniques reduces the number of S/R events (*p* < 0.001)	High
Garriga, M. [21]	Spain 2016	Clinical Practice Guidelines	55 observational studies, 39 RCT and 30 systematic reviews and metanalysis.	Differential aetiology, therapeutic environment; risk scales (OAS, ASS, BVC, HCR-20, and VSC); verbal de-escalation	The use of validated scales to measure the level of agitation and aggressive behavior was effective in most primary studies. The use of verbal de-escalation techniques is recommended given their potential to reduce anxiety and violence-associated risk.	High
Gaynes, B.N. [22]	USA 2017	Systematic review	21 studies, 3628 subjects	Staff training, risk scales, psychotherapies, sensory modulation, medication adjustment, complex programs	Risk assessment and multimodal programs were effective in decreasing aggressiveness and coercive measures	Medium
Goulet, M.H., et al. [24]	France 2017	Systematic review	23 studies (2 RCT)	“Six Core Strategies”, “Safewards”	Homogeneity of results. “Six Core Strategies” and “Safewards” are effective in reducing the mean use of coercive measures	Medium
Hirsch, S., [27]	2019	Systematic review	90 studies, 16 RCT	Staff training, organisation, risk scales, environment, debriefing, psychotherapy, advanced instructions, “Six Core Strategies”, “Safewards”	Staff training in de-escalation techniques, risk scales, reporting and discussing the measures with the patient, and advanced instructions; apart from “Six Core Strategies”, “Safewards”, these are useful in reducing the use of CM.	Medium
Patel, M.X., [32]	2018	Clinical Practice Guidelines	Systematic review, RCT and observational studies	Verbal de-escalation, offering medication, physical health monitoring.	All staff is advised to receive training in reducing the scale, and this is described as potentially useful to avoid MR and other types of restraint.	High
Rampling, J., [33]	2016	Systematic review	23 studies	Psychosocial therapies (CBT, R&R, DBT, ETS, ST, AAT)	There is certain evidence in reducing aggression during and after CBT (*p* < 0.05) for psychosis in male psychotic patients with an aggressive history. CBT, ETS, and R&R are well-tolerated, and there are improvements in the short and medium term. AAT is the only one with improvements in the long term.	Medium
Vakiparta, L., [36]	2019	Systematic review	28 studies (3 RCT)	Complex programs, therapeutic atmosphere, staff training, risk scales, Treatment planning, patient participation, sensory modulation, exclusive measures for the management of agitation.	Risk scales, sensory modulation, and patient participation were effective in reducing the use of MR. staff training was effective in reducing their duration. Complex restraint programs led to fewer events, lower duration and lower risk of MR.	Medium

*, quality assessment through EPHPP; AAT, animal-assisted therapy; ASS, agitation severity scale; BVC, Broset Violence Checklist; CAT, can assisted therapy; CBT, cognitive behavioral techniques; CI, confidence interval; CM, coercive measures; DASA, Dynamic Appraisal of Situational Aggression; DBT, dialectical behavior therapy; EAT, equine-assisted therapy; ETS, enhanced thinking skills; HCR-20, HCR-20 Scale for Assessing Risk of Violent Behavior; ICU, intensive care units; IG/CG, intervention/control group; MHHU, mental health hospitalization units; MR, mechanical restraints; OAS, overt aggression scale; OR, odds ratio; PMAV, prevention and management of aggression and violence; R&R, reasoning and rehabilitation; S/R, seclusion and restraint; VSC, violence screening checklist.

**Table 5:** Results of the methodological quality assessment (FLC 3.0) [38].

Study	Research Question	Method	Results	Conclusions	Conflicts of Interest	External Validity	Quality of the Study
	Is the study based on a clearly defined research question?	Has the method of study helped reduce biases?	Are the results adequately synthetised and described?	Are the conclusions justified?	Is the existence or lack of conflict of interest well described?	Are the study results applicable to the population and context of interest?	
Andersen, C. et al. [16]	Yes	Yes	Yes	Yes	Yes	Yes	High
Baldacara, L. et al. [17]	Yes	Yes	Yes	Yes	Yes	Yes	High
Bowers, L. et al. [19]	Yes	Yes	Yes	Yes	Yes	Yes	High
Dahm, K.T. et al. [20]	Yes	Yes	Yes	Yes	Yes	Partially	-
Gaynes, B.N. et al. [22]	Yes	Yes	Yes	Yes	-	Partially	Medium
Goulet, M.H. et al. [24]	Yes	Yes	Partially	Yes	Yes	Partially	Medium
Hirsch, S.; Steinert, T. [27]	Yes	Yes	Yes	Yes	Yes	Partially	Medium
Hvidhjelm, J. et al. [28]	Yes	Partially	Yes	Yes	Yes	Partially	Medium
Kaunomaki, J. et al. [29]	Yes	Partially	Yes	Yes	Yes	Partially	Medium
Nurenberg, J.R. et al. [30]	Yes	Yes	Yes	Yes	Yes	Yes	High
Rampling, J. et al. [33]	Partially	Yes	Yes	Partially	Yes	Partially	Medium
Schneeberger, A.R. et al. [34]	Yes	Partially	Yes	Yes	Yes	Partially	Medium
Vakiparta, L. et al. [36]	Yes	Partially	Yes	Yes	Yes	Partially	Medium
Suggestions from the F.L.C 3.0 system for assessment							
	Method area: yes		Method area: partially		Method area: No		
Majority rest of the areas: yes	High quality		Medium quality		Low quality		
Majority rest of the areas: partially	Medium quality		Low quality		Low quality		
Majority rest of the areas: No	Low quality		Low quality		Low quality		

Not assessable, having answered “no data” in the “Method” section or in the majority of areas, so it is not possible to assess the study quality.

**Table 6:** Results of the methodological quality assessment (Effective Public Health Practice Project, EPHPP) [39].

Study	Selection Bias	Study Design	Confusion Factors	Blind	Data Compilation Methods	Withdrawals and Abandonments	Overall Punctuation
Baumgardt, J. et al. [18]	1	2	2	3	2	2	2
Geoffrion, S. et al. [23]	2	2	2	3	1	2	2
Guay, S. et al. [25]	2	2	2	3			2
Guzman-Parra, J. et al. [26]	1	2	2	3	1	1	2
Long, C. et al. [31]	1	2	2	3	1	1	2
Stensgaard, L. et al. [35]	2	2	2	3	2	1	2

1: strong evidence; 2: medium evidence; 3: low evidence.
